# Cuproptosis in Inflammation-Related Respiratory Diseases

**DOI:** 10.3390/ijms27209218

**Published:** 2026-10-17

**Authors:** Qiaoli Fu, Yuanyuan Zheng, Yinchun Zou, Xinyi Cui, Rui Wei, Ying Gu, Lijun Qin, Shibo Sun

**Affiliations:** 1 First Department of Respiratory and Critical Care Medicine, The First Affiliated Hospital of Kunming Medical University, Kunming 650032, China; qiaolifu2002@163.com (Q.F.); zhengyuanyuan@ydyy.cn (Y.Z.); zyc33326@163.com (Y.Z.); yinggu0611@163.com (Y.G.); lijunqinky@163.com (L.Q.); 2 Department of Clinical Medicine, Kunming Medical University, Kunming 650500, China; xinyicui2027@163.com; 3 Department of Emergency Medicine, The First Affiliated Hospital of Kunming Medical University, Kunming 650032, China; weirui@kmmu.edu.cn

**Keywords:** cuproptosis, inflammation, respiratory diseases

## Abstract

Cuproptosis is a newly identified form of regulated cell death. The core molecular events of cuproptosis involve copper homeostasis dysregulation, aggregation of mitochondrial lipoylated proteins, loss of iron–sulfur cluster proteins, and mitochondrial proteotoxic stress. Multiple signaling pathways regulate cuproptosis, such as the mitogen-activated protein kinase (MAPK), hypoxia-inducible factor-1α (HIF-1α), and the p53 pathway. Cuproptosis plays an important role in various inflammation-related respiratory diseases, including lung cancer, acute lung injury/acute respiratory distress syndrome (ALI/ARDS), asthma, chronic obstructive pulmonary disease (COPD), and tuberculosis. Cuproptosis inhibits malignant progression and therapeutic resistance in lung cancer, while it aggravates ALI/ARDS. In addition, cuproptosis promotes airway inflammation and remodeling of asthma. However, the role of cuproptosis in COPD and tuberculosis remains unclear. Moreover, the mechanism of cuproptosis affecting inflammation-related respiratory diseases is unclear. Accordingly, cuproptosis may play an important role in various inflammation-related respiratory diseases.

## 1. Introduction

Cuproptosis is a novel form of regulated cell death discovered by Tsvetkov et al. in 2022 [1]. Cuproptosis is triggered by intracellular copper accumulation and is characterized by the aggregation of mitochondrial lipoylated proteins and the loss of iron–sulfur (Fe-S) cluster proteins [1,2]. It is reported that the aggregation of mitochondrial lipoylated proteins disrupts the tricarboxylic acid (TCA) cycle and oxidative phosphorylation, leading to mitochondrial dysfunction and excessive reactive oxygen species (ROS) generation to cause the cuproptosis [1,3]. In addition, the loss of Fe-S cluster proteins inhibits electron transport chain activity and cellular redox homeostasis, further amplifying the mitochondrial dysfunction, leading to the cuproptosis [4].

An increasing number of studies indicate that cuproptosis is closely related to inflammation [5,6]. On the one hand, pro-inflammatory cytokines trigger the canonical nuclear factor kappa B (NF-κB) pathway, playing a dual role in cuproptosis. NF-κB can increase the expression levels of copper transporter 1 (*CTR1/SLC31A1*), ultimately inducing intracellular copper overload and leading to cuproptosis [7]. However, NF-κB can also repress copper uptake by inhibiting the expression of *CTR1/SLC31A1* to suppress cuproptosis. In addition, cuproptosis amplifies inflammatory responses through the production of ROS, the release of damage-associated molecular patterns, and the activation of inflammatory pathways [7,8,9]. Moreover, mitochondrial dysfunction induced by cuproptosis creates a permissive environment for cuproptosis by depleting glutathione (GSH) and reducing the activity of superoxide dismutase 2 [10]. On the other hand, cuproptosis triggers pyroptosis in cells, thereby leading to pyroptosis-related inflammation [11,12]. Consequently, inflammation is a trigger of cuproptosis, and cuproptosis in turn exacerbates inflammation.

Respiratory diseases are major global causes of mortality and morbidity [13]. Inflammation plays an important role in several respiratory diseases, including pneumonia, lung cancer, acute lung injury/acute respiratory distress syndrome (ALI/ARDS), chronic obstructive pulmonary disease (COPD), tuberculosis, and asthma [12,14]. Accumulating evidence suggests that cuproptosis plays an important role in inflammation-related respiratory diseases and may be a therapeutic target for various inflammation-related respiratory diseases. This article aimed to review the role and molecular mechanisms of cuproptosis in inflammation-related respiratory diseases.

## 2. Molecular Mechanisms of Cuproptosis

### 2.1. Copper Homeostasis Dysregulation

Copper is an essential trace element in humans and plays a critical role in various physiological processes. Both excess or deficiency of copper impairs cellular function and leads to cell death in severe cases [15]. Copper homeostasis is dynamically maintained by copper homeostasis-related proteins, including CTR1/SLC31A1, cytochrome c oxidase copper chaperone (COX17), antioxidant 1 copper chaperone (ATOX1), copper chaperone for superoxide dismutase (CCS), copper-ion-transporting ATPases alpha (ATP7A), and copper-ion-transporting ATPases beta (ATP7B) [16]. Excess intracellular copper accumulation, particularly within mitochondria, disrupts metabolic processes and promotes proteotoxic stress, ultimately leading to cuproptosis [17].

**Figure 1:**
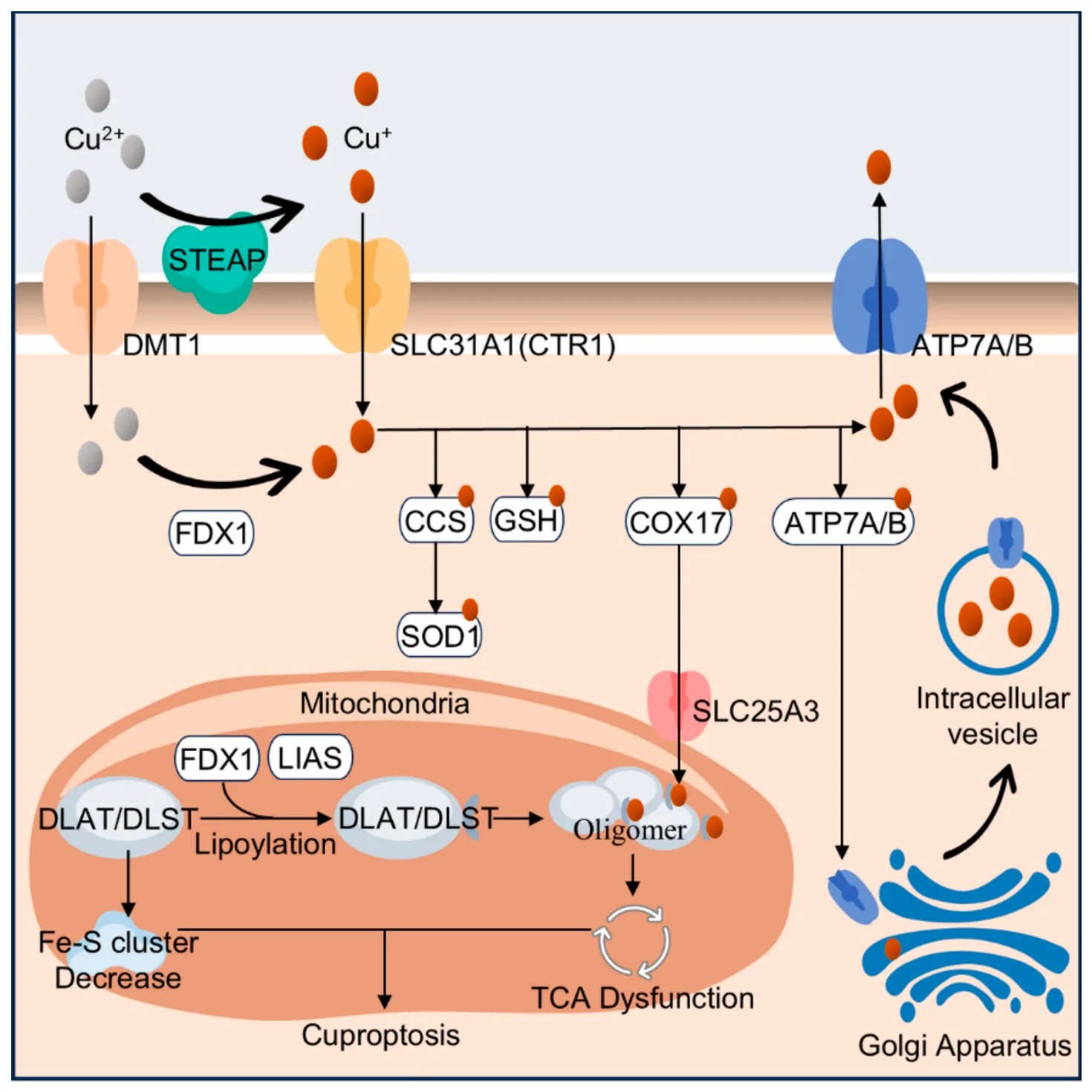
The mechanism of cuproptosis. Extracellular Cu^2+^ is imported into cells via DMT1 and reduced to Cu^+^ by STEAP. Cu^+^ subsequently enters the cytoplasm through CTR1/SLC31A1. Intracellular Cu^+^ is distributed to multiple pathways. FDX1 mediates copper reduction and is essential for mitochondrial protein lipoylation. CCS delivers copper to SOD1. GSH chelates excess copper for detoxification. COX17 shuttles copper into mitochondria via SLC25A3, and ATP7A/B mediates copper export by releasing vesicles through the Golgi apparatus. Within mitochondria, FDX1 and LIAS catalyze the lipoylation of DLAT and DLST, which is required for pyruvate dehydrogenase complex activity. Excess copper induces oligomerization of lipoylated DLAT/DLST, resulting in loss of Fe-S cluster proteins, TCA cycle dysfunction, and ultimately cuproptosis. Abbreviations: DMT1, divalent metal transporter 1; STEAP, six-segment transmembrane epithelial antigen of prostate; CTR1/SLC31A1, copper transporter 1; ATP7A/B, copper-ion-transporting ATPases alpha/beta; FDX1, ferredoxin 1; CCS, copper chaperone for superoxide dismutase; GSH, glutathione; COX17, cytochrome c oxidase copper chaperone; SOD1, superoxide dismutase 1; LIAS, lipoic acid synthetase; SLC25A3, solute carrier family 25 member 3; DLAT, dihydrolipoamide acetyltransferase; DLST, dihydrolipoamide succinyltransferase; Fe-S, iron–sulfur; TCA, tricarboxylic acid.

Copper exists in either reduced (Cu^+^) or oxidized (Cu^2+^) state, and redox properties of copper make it both beneficial and toxic to cells [18]. Free intracellular copper generates ROS through the Fenton-like reaction (Cu^+^ + H_2_O_2_ → Cu^2+^ + OH^−^ + OH) [19]. ROS can damage the structure of DNA, destroy the mitochondrial inner membrane, and increase the permeability of cell membranes, thereby inducing cuproptosis [20,21,22,23,24,25]. In addition, GSH protects against cuproptosis through chelating excess copper and scavenging ROS [26,27]. Once GSH is exhausted during cuproptosis, the cell removes the buffering capacity, thereby lowering the threshold for cuproptosis (Figure 1).

Accordingly, copper homeostasis dysregulation is the initiating event of cuproptosis.

### 2.2. Aggregation of Mitochondrial Lipoylated Proteins

Lipoylated proteins are mitochondrial enzymes covalently modified by lipoic acid (LA) on specific lysine residues, functioning as essential cofactors for acyl and electron transfer in energy metabolism [28,29]. Notably, cuproptosis primarily occurs in cells with active oxidative phosphorylation, where abundant lipoylated proteins are present [1]. Within mitochondria, ferredoxin 1 (FDX1) reduces Cu^2+^ to Cu^+^ [30]. Subsequently, Cu^+^ directly binds to the lipoyl moiety of lipoylated TCA enzymes, resulting in the aggregation of lipoylated proteins [31]. Dihydrolipoamide acetyltransferase (DLAT) and dihydrolipoamide succinyltransferase (DLST) are the primary lipoylated proteins to be aggregated [32,33,34,35]. This process induces the production of heat shock protein 70 (HSP70) and triggers proteotoxic stress [3]. Moreover, the aggregation of lipoylated proteins directly inactivates the pyruvate dehydrogenase complex, inhibiting the TCA cycle and oxidative phosphorylation, ultimately causing a sharp decline in ATP synthesis and metabolic dysregulation [36,37]. The resulting mitochondrial dysfunction is accompanied by a burst of ROS, ultimately inducing cuproptosis [38]. Collectively, the aggregation of lipoylated proteins is an upstream central event that triggers cuproptosis.

### 2.3. Loss of Fe-S Cluster Proteins

Fe-S cluster proteins are essential cofactors for many key enzymes in the mitochondrial electron transport chain complexes and the TCA cycle [39,40,41]. The collapse of the electron transport chain exacerbates electron leakage, leading to an explosive production of ROS and causing severe oxidative stress [42]. Moreover, the loss of Fe-S cluster proteins severely impairs mitochondrial respiration, leading to a cellular energy crisis [42,43]. Studies have shown that excess Cu^+^ competes with Fe^2+^ binding to iron–sulfur cluster assembly 1/2 (ISCA1/2) in mitochondria, disrupting the formation of Fe-S cluster proteins [44,45]. Additionally, copper-triggered aggregation of lipoylated proteins suppresses the formation of Fe-S cluster proteins and promotes the degradation of Fe-S cluster proteins, subsequently triggering proteotoxic stress and cuproptosis [45,46,47]. Notably, disruption of Fe-S cluster proteins in turn impairs the formation of lipoylated proteins [32]. Nevertheless, pre-existing lipoylated proteins remain susceptible to aggregation under copper overload, forming a positive feedback loop to exacerbate cuproptosis [1]. Accordingly, loss of Fe-S cluster proteins is a downstream amplifier to promote cuproptosis.

### 2.4. Mitochondrial Proteotoxic Stress

The aggregation of lipoylated proteins and the loss of Fe-S cluster proteins collectively induce proteotoxic stress [3,48]. Research indicates that aggregation of mitochondrial lipoylated proteins alters cristae organization, thereby inducing proteotoxic stress [49]. The upregulated heat shock proteins (HSPs), particularly HSP70, improve protein misfolding and aggregation and sends them to ubiquitin–proteasome system (UPS)-degradation or lysosome-autophagy-degradation pathways to avoid cell damage [50,51]. However, the aggregation of lipoylated proteins rapidly overwhelms the chaperone capacity, ultimately resulting in collapse of matrix protein homeostasis [52]. Moreover, severe mitochondrial dysfunction induced by lipoylated protein aggregation and Fe-S cluster protein loss causes the release of mtDNA into the cytoplasm to activate the cGAS-STING pathway, further aggravating cuproptosis [53,54,55].

In addition, the copper homeostasis dysregulation indirectly influences mitochondrial proteotoxic stress. The 20S proteasome is the catalytic engine of the UPS to maintain protein homeostasis by degrading intracellular proteins [20,56]. However, copper directly inhibits the 20S proteasome, reducing cellular protease activity, blocking cell growth, and leading to cuproptosis [57].

Collectively, mitochondrial proteotoxic stress caused by the aggregation of lipoylated proteins and the loss of Fe-S cluster proteins is the typical feature of cuproptosis. In addition, cuproptosis can be experimentally identified by lipoylated protein aggregation, loss of Fe-S cluster proteins, and elevated intracellular copper [51]. However, due to the lack of a specific single marker for cuproptosis, multiple indicators need to be combined for judgment in the identification of cuproptosis.

## 3. Signaling Pathways of Cuproptosis

**Figure 2:**
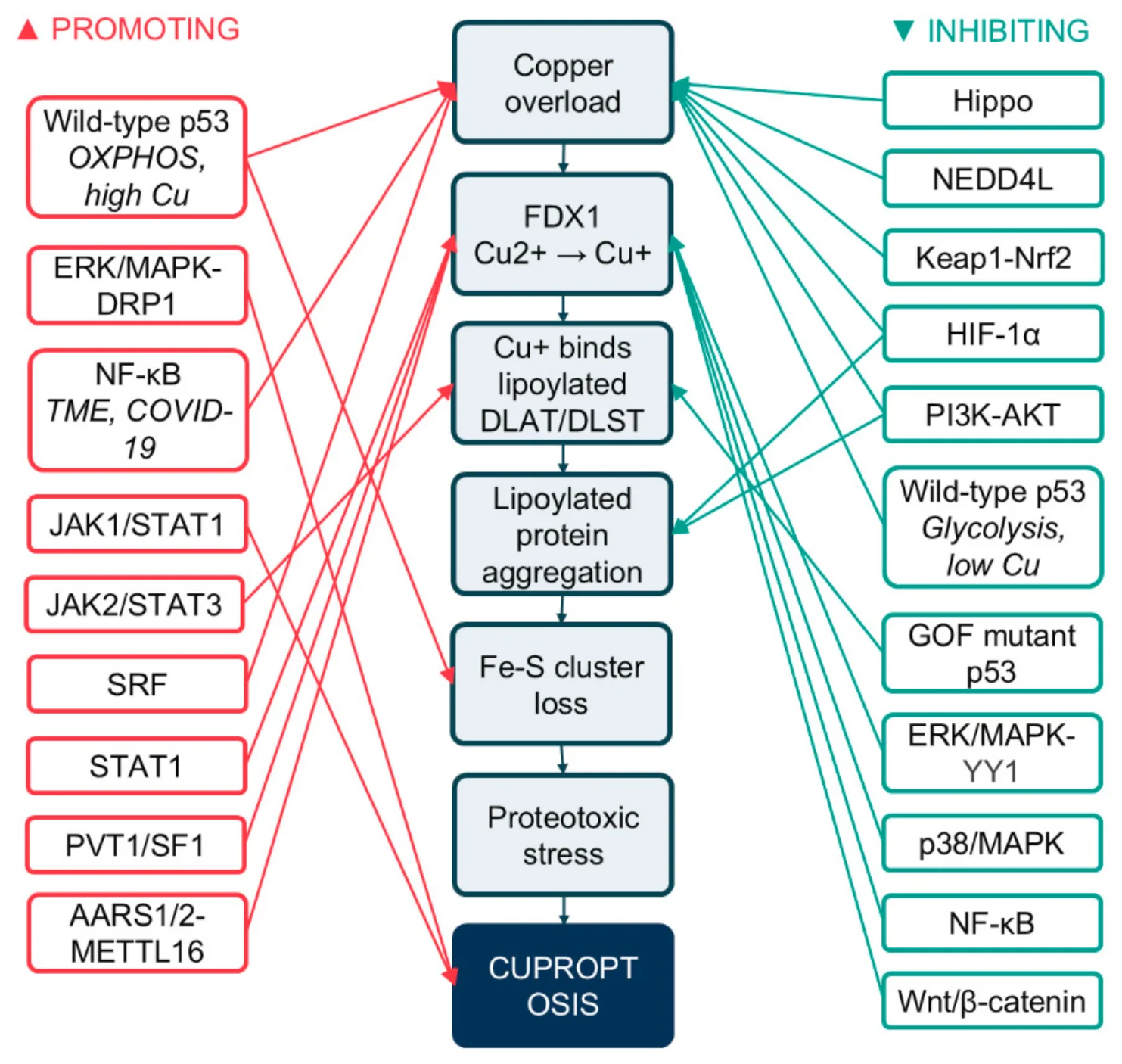
The core signaling pathways of cuproptosis. Copper overload drives FDX1-mediated reduction of Cu^2+^ to Cu^+^. Subsequently, Cu^+^ binds to lipoylated DLAT/DLST, triggering lipoylated protein aggregation, loss of Fe–S cluster proteins, and proteotoxic stress, ultimately leading to cuproptosis. Signaling pathways that promote (red, left) or inhibit (green, right) cuproptosis are indicated. Abbreviations: OXPHOS, oxidative phosphorylation; ERK, extracellular signal-regulated kinase; MAPK, mitogen-activated protein kinase; DRP1, dynamin-related protein 1; NF-κB, nuclear factor kappa B; TME, tumor microenvironment; JAK1, Janus kinase 1; STAT1, signal transducer and activator of transcription 1; JAK2, Janus kinase 2; STAT3, signal transducer and activator of transcription 3; SRF, serum response factor; PVT1, plasmacytoma variant translocation 1; SF1, splicing factor 1; AARS1/2, alanyl-tRNA synthetase 1/2; METTL16, methyltransferase-like 16; FDX1, ferredoxin 1; DLAT, dihydrolipoamide acetyltransferase; DLST, dihydrolipoamide succinyltransferase; Fe-S, iron–sulfur; NEDD4L, neural precursor cell expressed developmentally downregulated 4-like; Keap1, Kelch-like ECH-associated protein 1; Nrf2, nuclear factor erythroid 2-related factor 2; HIF-1α, hypoxia-inducible factor-1α; PI3K, phosphoinositide 3-kinase; AKT, protein kinase B; GOF, gain-of-function; YY1, Yin Yang 1.

Cuproptosis is regulated by multiple signaling pathways, including the MAPK pathway, HIF-1α pathway, p53 pathway, JAK/STAT pathway, NF-κB pathway, PI3K-AKT pathway, Hippo pathway, Wnt/β-catenin pathway, Keap1-Nrf2 pathway, and other regulatory factors of cuproptosis (Figure 2).

### 3.1. Keap1-Nrf2 Signaling Pathway

The Kelch-like ECH-associated protein 1–nuclear factor erythroid 2-related factor 2 (Keap1-Nrf2) signaling pathway plays a critical role in protecting cells against oxidative stress [58]. Under copper overload, Keap1 undergoes conformational changes, leading to Nrf2 degradation and nuclear translocation [59,60]. In the nucleus, Nrf2 subsequently binds to antioxidant response elements, resulting in copper homeostasis dysregulation and preventing the aggregation of lipoylated proteins, thereby inhibiting cuproptosis [61,62,63].

### 3.2. HIF-1α Signaling Pathway

The hypoxia-inducible factor-1α (HIF-1α) signaling pathway is a critical regulator of cellular metabolic reprogramming. Under hypoxia, the activity of prolyl hydroxylase domain proteins is suppressed, inhibiting HIF-1α degradation, leading to stabilization and accumulation of HIF-1α. Subsequently, HIF-1α translocates into the nucleus to heterodimerize with aryl hydrocarbon receptor nuclear translocator (ARNT), thereby forming the HIF-1α/ARNT complex [64]. The HIF-1α/ARNT complex binds to hypoxia response elements, thereby upregulating the pyruvate dehydrogenase kinase 1/3 (*PDK1/3*) [64,65]. Then the activated PDK1/3 promotes the ubiquitin-mediated degradation of DLAT, thereby inhibiting cuproptosis [66,67]. In addition, the HIF-1α/ARNT complex binds to hypoxia response elements to induce the expression of metallothionein 2A, which regulates copper homeostasis to inhibit cuproptosis [66,67].

### 3.3. PI3K-AKT Signaling Pathway

The phosphoinositide 3-kinase-protein kinase B (PI3K-AKT) signaling pathway is a central regulator of cell survival, metabolism, and stress adaptation [68]. Copper activates PI3K to promote the conversion of phosphatidylinositol 4,5-bisphosphate (PIP2) to phosphatidylinositol (3,4,5)-trisphosphate (PIP3) [69]. PIP3 subsequently recruits and activates AKT to activate or inhibit effect proteins in the nucleus [70]. Collectively, the PI3K-AKT pathway is activated. Subsequently, the activated PI3K-AKT pathway inhibits cuproptosis by dysregulating copper homeostasis and preventing the aggregation of lipoylated proteins [71,72,73].

### 3.4. p53 Signaling Pathway

Multiple forms of p53 protein exist in mammalian cells, including wild-type p53, gain-of-function mutant p53, loss-of-function/null p53, and alternative-splicing isoforms. Among them, wild-type p53 and gain-of-function mutant p53 have been reported to be associated with cuproptosis [74].

Upon high levels of copper, wild-type p53 is activated and subsequently translocates into the nucleus [74]. Thus, the wild-type p53 pathway is activated. It is reported that the wild-type p53 pathway may participate in cuproptosis in a context-dependent manner, which is jointly regulated by cellular metabolic state, types of stress stimuli, and intracellular copper burden [74]. In oxidative phosphorylation-predominant cells under high copper overload, wild-type p53 promotes cuproptosis by reducing GSH biosynthesis and inducing Fe-S cluster proteins biogenesis [74,75,76]. In contrast, in glycolysis-biased cells or under low copper stress, wild-type p53 may prevent cuproptosis by promoting the production of GSH [74]. Different from wild-type p53, gain-of-function mutant p53 may reduce the lipoylated protein for Cu^+^ binding, thereby reducing lipoylated protein aggregation and inhibiting cuproptosis [74].

In addition, p53 may disrupt Fe-S cluster proteins biogenesis by impairing the mitochondrial function of HSPA9, thereby inhibiting cuproptosis [77,78]. However, authors suggest that the loss of Fe-S cluster proteins generally leads to cuproptosis [79]. This contradiction indicates that the roles of cuproptosis still require further verification in the p53 pathway.

### 3.5. MAPK Signaling Pathway

The mitogen-activated protein kinase (MAPK) signaling pathway mainly includes the extracellular signal-regulated kinase (ERK)/MAPK signaling pathway and the p38-MAPK signaling pathway. The MAPK pathway plays an important role in cell death and stress responses through MAPK cascade [80,81].

In the ERK/MAPK pathway, copper ions activate the ERK/MAPK pathway [82,83,84]. It is reported that the ERK/MAPK pathway plays dual roles in cuproptosis. The ERK/MAPK pathway inhibits cuproptosis by upregulating Yin Yang 1 (*YY1*), while it promotes cuproptosis by upregulating dynamin-related protein 1 (DRP1) [85,86].

In the p38-MAPK pathway, p38-MAPK inhibits cuproptosis by phosphorylating forkhead box O3 (FOXO3) [87,88,89].

### 3.6. NF-κB Signaling Pathway

Upon stimulation by pro-inflammatory cytokines, oxidative stress, and copper overload, TGF-beta-activated kinase 1 (TAK1) is activated to phosphorylate and activate the inhibitor of κB kinase (IKK) [90]. Activated IKK subsequently phosphorylates IκB, thereby triggering IκB ubiquitination and proteasomal degradation. The degraded IκB releases NF-κB to trigger the nuclear translocation of NF-κB; thus, the NF-κB signaling pathway is activated.

The NF-κB signaling pathway plays a dual role in cuproptosis by regulating copper homeostasis. On the one hand, the NF-κB pathway induces intracellular copper overload in tumor microenvironments, ultimately leading to cuproptosis [7]. On the other hand, the activation of the NF-κB pathway inhibits cuproptosis by reducing the intracellular level of copper in breast cancer models [90,91,92]. The role of the NF-κB pathway in cuproptosis may vary with different cell types and diseases. Accordingly, the precise mechanisms causing these opposing regulatory effects remain unclear.

Furthermore, the NF-κB pathway inhibits cuproptosis by suppressing the *FDX1* and *DLAT* [93,94,95].

### 3.7. Wnt/β-Catenin Signaling Pathway

The Wnt combines with Frizzled and low-density lipoprotein receptor-related protein 5/6 (LRP5/6) to form a dimerization, thereby inhibiting the degradation of β-catenin [96]. Subsequently, β-catenin accumulates and transports into the nucleus, leading to the activation of the Wnt/β-catenin signaling pathway. The Wnt/β-catenin pathway inhibits cuproptosis by forming a transcriptional complex with transcription factor 4 (TCF4) [97,98]. In addition, the Wnt/β-catenin pathway inhibits cuproptosis by reducing *FDX1* and lipoic acid synthetase (*LIAS*) [99,100,101].

### 3.8. Hippo Signaling Pathway

When the Hippo signaling pathway is activated, mammalian sterile 20-like kinases 1/2 (MST1/2) activate large tumor suppressor kinases 1/2 (LATS1/2) [102]. Subsequently, LATS1/2 phosphorylates Yes-associated protein/transcriptional coactivator with PDZ-binding motif (YAP/TAZ) [102]. The phosphorylated YAP/TAZ is degraded to maintain intracellular copper homeostasis, inhibiting cuproptosis [103,104]. When the Hippo pathway is inhibited, the degradation of YAP/TAZ is inhibited to result in YAP/TAZ accumulation and nuclear translocation [103]. Within the nucleus, YAP/TAZ dysregulates cellular copper homeostasis, leading to cuproptosis [104,105,106].

### 3.9. JAK/STAT Signaling Pathway

The Janus kinase/signal transducer and activator of transcription (JAK/STAT) signaling pathway is an important regulator of cuproptosis and mainly includes the JAK1/STAT1 signaling pathway and the JAK2/STAT3 signaling pathway.

Upon binding of interferon-γ (IFN-γ), JAK1 phosphorylates and activates STAT1. Activated STAT1 translocates into the nucleus and induces interferon regulatory factor 1 (IRF1) to promote cuproptosis [107].

In addition, inflammation triggers JAK2 to phosphorylate STAT3 [108,109]. Thus, the JAK2/STAT3 pathway is activated. The JAK2/STAT3 pathway leads to cuproptosis by driving cytoplasmic copper trafficking into the mitochondrial matrix [109].

### 3.10. Other Regulatory Factors of Cuproptosis

There are other regulatory factors in cuproptosis. It is reported that the serum response factor (SRF) and STAT1 promote cuproptosis by upregulating *CTR1/SLC31A1*, whereas neural precursor cell expressed developmentally downregulated 4-like (NEDD4L) suppresses cuproptosis by degrading CTR1/SLC31A1 [110,111,112]. Meanwhile, multiple regulatory factors promote cuproptosis by elevating *FDX1*, including plasmacytoma variant translocation 1/splicing factor 1 (*PVT1/SF1*) and alanyl-tRNA synthetase 1/2-methyltransferase-like 16 (*AARS1/2-METTL16*) [113,114].

## 4. Cuproptosis and Inflammation-Related Respiratory Diseases

Cuproptosis is related to various inflammation-related respiratory diseases, such as pneumonia, lung cancer, ALI/ARDS, COPD, tuberculosis, and asthma, and multiple signaling pathways are involved.

### 4.1. Cuproptosis and Pneumonia

Pneumonia is one of the most challenging infectious diseases worldwide with high morbidity and mortality rates. Bacterial, viral, and fungal pathogens trigger robust inflammatory responses in the lung, leading to alveolar–capillary barrier disruption, inflammatory cell infiltration, and impaired gas exchange [115]. Moreover, according to the setting of acquisition, pneumonia can be classified into community-acquired pneumonia (CAP) and hospital-acquired pneumonia. It is reported that cuproptosis plays an important role in bacterial pneumonia and CAP.

Cuproptosis is a promising antibacterial strategy against antibiotic-resistant bacterial pneumonia. It is reported that although bacteria lack mitochondria, bacteria can be affected by copper toxicity resulting in lipoylated protein aggregation and loss of Fe-S cluster proteins, which may cause cuproptosis [116]. This cell death is termed cuproptosis-like death in bacteria [116]. Notably, amplified cuproptosis-like death in bacteria can alleviate pneumonia-associated pathological changes [117,118].

In addition, multiple cuproptosis-related genes (CRGs) are differentially expressed in severe CAP, including *ATP7B*, dihydrolipoamide branched chain transacylase E2 (*DBT*), *DLAT*, dihydrolipoamide dehydrogenase (*DLD*), *FDX1*, glycine cleavage system protein H (*GCSH*), LIAS, lipoyltransferase 1 (*LIPT1*), and *CTR1/SLC31A1* [119,120]. These differentially expressed CRGs in severe CAP may be associated with immune cell infiltration and have an important role in the immunopathology of pneumonia [119]. However, the specific roles of these CRGs and cuproptosis remain unclear in CAP.

Accordingly, cuproptosis may play a crucial role in bacterial pneumonia and CAP.

### 4.2. Cuproptosis and Lung Cancer

Lung cancer is one of the leading causes of cancer-related mortality worldwide, primarily driven by cigarette exposure and air pollution [121,122,123].

Copper homeostasis plays an important role in cuproptosis of lung cancer. In lung cancer, serum copper levels are elevated and correlate with cancer progression [124,125]. Meanwhile, accumulated copper induces cuproptosis in lung cancer [126].

Multiple signaling pathways regulate cuproptosis to mediate malignant progression, metastasis, and therapeutic resistance in lung cancer, including the Keap1-Nrf2, HIF-1α, PI3K-AKT, and p53 pathways. In lung cancer, the Keap1-Nrf2 pathway upregulates GSH synthesis, reducing cuproptosis [127]. Meanwhile, the HIF-1α pathway mediates resistance mechanisms by suppressing cuproptosis in non-small cell lung cancer (NSCLC) [128]. In lung adenocarcinoma (LUAD), the PI3K-AKT pathway induces cuproptosis to inhibit cancer growth [129,130]. In contrast, the p53 pathway promotes cuproptosis to inhibit the progression of LUAD [131].

Current studies on CRGs are highly concentrated in LUAD, with only sporadic reports in lung squamous cell carcinoma (LUSC) and almost no reports in small cell lung cancer (SCLC) or other subtypes. Studies on cuproptosis in SCLC, large-cell lung carcinoma, and neuroendocrine tumors remain insufficient. In LUAD, 10 CRGs are differentially expressed [132,133,134]. Among them, high *DLD* expression and low *LIPT1* expression were each significantly associated with shorter overall survival [134]. Pyruvate dehydrogenase E1 subunit alpha 1 (*PDHA1*) has been proposed as a predictive biomarker for immunotherapy response across cancers, with its expression also associated with overall survival in LUAD [133]. Notably, *FDX1* is a core regulator of cuproptosis [132]. The overexpression of *FDX1* may inhibit tumor cell proliferation, migration, and invasion by the G protein-regulated inducer of neurite outgrowth 2 (GPRIN2)/PI3K signaling pathway. In LUSC, *NFE2L2* (the gene encoding Nrf2), *LIPT1*, NOD-like receptor thermal protein domain associated protein 3 (*NLRP3*), glutaminase (*GLS*), *LIPT2*, *DLD*, metal regulatory transcription factor 1 (*MTF1*), *PDHA1* and *LIAS* are amplified in most patients, while *FDX1*, *ATP7B*, *DLAT*, *GCSH*, *DBT*, pyruvate dehydrogenase E1 subunit beta (*PDHB*) and cyclin dependent kinase inhibitor 2A (*CDKN2A*) are absent [133]. By studying the effect of gene expression patterns on the overall survival rate of LUSC, it was found that patients with high expression of *ATP7B*, *DLAT*, *DLST*, *GLS* and *NLRP3* had poor prognosis, while patients with high expression of *ATP7A*, *DLD*, *LIPT1*, *NFE2L2* and *PDHA1* had improved prognosis [133]. However, the specific functions of the CRGs in lung cancer currently rely on bioinformatic speculation. Moreover, these conclusions have not been verified through experiments.

Moreover, cuproptosis amplifies immunotherapy efficiency [135,136,137]. Accordingly, cuproptosis may play important roles in lung cancer. However, the specific mechanisms of cuproptosis in lung cancer remain to be further elucidated.

### 4.3. Cuproptosis and ALI/ARDS

ALI/ARDS is a life-threatening hypoxemic respiratory disease caused by multiple pathogenic factors, and is characterized by hypoxemia, diffuse bilateral pulmonary infiltration, and pulmonary edema [138].

Malaria-associated acute lung injury/acute respiratory distress syndrome (MA-ALI/ARDS) is a fatal complication of Plasmodium falciparum infection that is partially triggered by macrophage recruitment and polarization [139]. In MA-ALI/ARDS, the level of cuproptosis is enhanced and strongly associated with the severity of MA-ALI/ARDS [139]. The activation of cuproptosis induces macrophage M1 polarization to aggravate MA-ALI/ARDS, whereas inhibition of cuproptosis induces macrophage M2 polarization and reduces macrophage M1 polarization to alleviate MA-ALI/ARDS [138,139]. Notably, distinct from non-MA-ALI/ARDS, the pathogenesis of MA-ALI/ARDS is a monocyte-dominant inflammatory response rather than neutrophils. Studying the role of cuproptosis in MA-ALI/ARDS will help to reveal the specific mechanism of cuproptosis in different subtypes of ALI/ARDS.

Sepsis is the most common cause of ALI/ARDS. In sepsis-related ALI/ARDS, cuproptosis disrupts the endothelial barrier through mitochondrial dysfunction and excessive ROS, ultimately leading to vascular leakage, pulmonary edema, and hypoxemia [140]. Notably, vascular leakage, pulmonary edema, and hypoxemia are the key pathological features of ALI/ARDS [140].

The coronavirus disease 2019 (COVID-19), caused by severe acute respiratory syndrome coronavirus 2 (SARS-CoV-2), is an important viral cause of ALI/ARDS [141]. It is reported that serum copper levels are elevated in COVID-19 and are correlated with disease severity [142,143,144]. Moreover, the SARS-CoV-2 spike protein, an important protein in SARS-CoV-2, induces intracellular copper accumulation in alveolar epithelial cells via the NF-κB pathway, subsequently triggering cuproptosis [145]. Furthermore, a cuproptosis-related risk score, based on *PDHA1*, *PDHB*, *MTF1* and *CDKN2A*, and a nomogram were created, which both showed excellent predictive values for COVID-19 [141].

Collectively, cuproptosis may play an important role in ALI/ARDS.

### 4.4. Cuproptosis and Tuberculosis

Tuberculosis is a chronic inflammatory disease caused by mycobacterium tuberculosis (Mtb) and is characterized by caseous necrotizing granuloma formation [146]. In tuberculosis, copper homeostasis plays a dual role in host–pathogen interactions. Host macrophages utilize copper against Mtb, whereas Mtb has evolved multiple resistance mechanisms to counteract copper toxicity [147,148,149]. Notably, Mtb can stimulate cuproptosis of macrophages, subsequently promoting macrophage death and bacterial spread [150].

In addition, 11 CRGs involved in cuproptosis are abnormally expressed in tuberculosis. Among tuberculosis patients, *NFE2L2*, *NLRP3*, *ATP7B*, *CTR1/SLC31A1*, *MTF1*, and *DLD* are significantly upregulated, whereas *LIAS*, *LIPT1*, *DLAT*, *GLS*, and *DBT* are downregulated [151]. The abnormal expression of *NFE2L2* is associated with susceptibility to tuberculosis. Additionally, emerging evidence suggests that Mtb could cause damage to alveolar epithelial cells, while *GLS* is necessary for alveolar epithelial regeneration. However, whether the other genes are involved in the pathogenesis of tuberculosis remains unclear.

Since tuberculosis predominantly manifests in immunocompromised hosts, the interplay between cuproptosis and the weakened immune system needs further research. Collectively, cuproptosis may be related to tuberculosis. However, the precise mechanisms of cuproptosis in tuberculosis need further investigation.

### 4.5. Cuproptosis and COPD

COPD is one of the leading causes of morbidity and mortality worldwide and is characterized by persistent airway inflammation, progressive airflow limitation, and alveolar destruction [152,153].

It is reported that the level of copper is elevated in COPD, which is strongly correlated with pulmonary inflammation [154]. Moreover, the elevated copper is a trigger of cuproptosis. Thus, copper-triggered cuproptosis may occur in COPD. Additionally, the expression of CRGs is abnormal in COPD, including *GLS*, *DLD*, *CDKN2A*, *DLST*, *LIPT1*, *MTF1*, and *PDHB* [155,156,157]. Among these, *GLS* expression is significantly reduced in COPD and correlates with the grade of pulmonary function decline, particularly in alveolar macrophages. Furthermore, *DLD* and *CDKN2A* are upregulated in response to smoking, and their aberrant expression is significantly correlated with lung function grading, suggesting their potential involvement in cuproptosis [156].

Accordingly, cuproptosis may be involved in COPD. Notably, most of the research on cuproptosis and COPD is mainly related to tobacco-smoking COPD. Currently, there is no direct evidence regarding whether cuproptosis is involved in occupational COPD. The specific role of cuproptosis requires further functional validation in COPD.

### 4.6. Cuproptosis and Asthma

Asthma is a global health burden and characterized by chronic airway inflammation, airway hyperresponsiveness, and airway remodeling [158]. It is reported that cuproptosis induces the increased airway responsiveness, lung inflammation, area of subepithelial fibrosis, mucus hypersecretion, and expression of epithelial–mesenchymal transition-related proteins, thereby promoting airway remodeling in asthma [159,160]. However, inhibiting cuproptosis can reverse the above process [159]. Accordingly, cuproptosis may be involved in the pathogenesis of asthma. However, the signaling pathways in which copper is involved in asthma remain unclear and require further investigation. Additionally, the function of cuproptosis in different asthma endotypes remain unclear, such as Th2-high and Th2-low.

## 5. Conclusions

Cuproptosis is a newly identified form of regulated cell death driven by intracellular copper accumulation, which is characterized by aggregation of lipoylated proteins, loss of Fe-S cluster proteins, and proteotoxic stress. Multiple signaling pathways regulate cuproptosis, including the MAPK pathway, HIF-1α pathway, p53 pathway, JAK/STAT pathway, NF-κB pathway, PI3K-AKT pathway, Hippo pathway, Wnt/β-catenin pathway, Keap1-Nrf2 pathway, and other regulatory factors of cuproptosis.

The cuproptosis is related to inflammation-related respiratory diseases, including pneumonia, lung cancer, ALI/ARDS, COPD, tuberculosis, and asthma. In pneumonia, cuproptosis-like death of bacteria alleviates pneumonia. In lung cancer, multiple signaling pathways regulate cuproptosis to mediate malignant progression and therapeutic resistance, including the Keap1-Nrf2, HIF-1α, PI3K-AKT, and p53 pathways. In ALI/ARDS, cuproptosis aggravates disease severity through macrophage M1/M2 polarization and disrupts the endothelial barrier. In tuberculosis, host macrophages utilize copper against Mtb, whereas Mtb stimulates macrophage cuproptosis to promote bacterial spread. In COPD, the abnormal expression of CRGs serves as a diagnostic biomarker, although their precise role remains unclear. In asthma, cuproptosis is enhanced and associated with airway inflammation and remodeling, yet its underlying mechanisms require further investigation.

However, there are several challenges in the relationship between cuproptosis and inflammation-related respiratory diseases. Firstly, most current evidence is derived from the results of bioinformatic analyses, cell models, or animal studies, whereas clinical validation remains limited. Secondly, cuproptosis exerts distinct biological effects across different tissues and disease states. Meanwhile, the therapeutic value of targeting cuproptosis may vary with different inflammation-related respiratory diseases, cell types, and pathological stages. Thirdly, the specific mechanisms and related signaling pathways of cuproptosis in inflammation-related respiratory diseases remain unclear.

Collectively, cuproptosis may play an important role in various inflammation-related respiratory diseases. Furthermore, targeting cuproptosis holds therapeutic promise for inflammation-related respiratory diseases. It is reported that copper ionophores and copper chelators have been used to regulate cuproptosis and have achieved certain therapeutic effects. These cuproptosis regulators have been explored mainly in cancer models, whereas their application in inflammation-related respiratory diseases remains largely unexplored. Moreover, the therapeutic value of targeting cuproptosis may vary with different diseases, cell types, and pathological stages. Thus, the direction and molecular targets of targeting cuproptosis must be defined in specific diseases and cell-types and need further investigation.

## Data Availability

No new data were created or analyzed in this study. Data sharing is not applicable to this article.

## Abbreviations

The following abbreviations are used in this manuscript:

Fe-S	Iron–sulfur
TCA	Tricarboxylic acid
ROS	Reactive oxygen species
NF-κB	Nuclear factor kappa B
CTR1/SLC31A1	Copper transporter 1
GSH	Glutathione
ALI/ARDS	Acute lung injury/acute respiratory distress syndrome
COPD	Chronic obstructive pulmonary disease
COX17	Cytochrome c oxidase copper chaperone
ATOX1	Antioxidant 1 copper chaperone
CCS	Copper chaperone for superoxide dismutase
ATP7A	Copper-ion-transporting ATPases alpha
ATP7B	Copper-ion-transporting ATPases beta
LA	Lipoic acid
FDX1	Ferredoxin 1
DLAT	Dihydrolipoamide acetyltransferase
DLST	Dihydrolipoamide succinyltransferase
HSP70	Heat shock protein 70
ISCA1/2	Iron–sulfur cluster assembly 1/2
HSPs	Heat shock proteins
UPS	Ubiquitin–proteasome system
Keap1-Nrf2	Kelch-like ECH-associated protein 1–nuclear factor erythroid 2-related factor 2
HIF-1α	Hypoxia-inducible factor-1α
ARNT	Aryl hydrocarbon receptor nuclear translocator
PDK1/3	Pyruvate dehydrogenase kinase 1/3
PI3K-AKT	Phosphoinositide 3-kinase-protein kinase B
PIP2	Phosphatidylinositol 4,5-bisphosphate
PIP3	Phosphatidylinositol (3,4,5)-trisphosphate
MAPK	Mitogen-activated protein kinase
ERK	Extracellular signal-regulated kinase
YY1	Yin Yang 1
DRP1	Dynamin-related protein 1
FOXO3	Forkhead box O3
TAK1	TGF-beta-activated kinase 1
IKK	Inhibitor of κB kinase
LRP5/6	Low-density lipoprotein receptor-related protein 5/6
TCF4	Transcription factor 4
LIAS	Lipoic acid synthetase
MST1/2	Mammalian sterile 20-like kinases 1/2
LATS1/2	Large tumor suppressor kinases 1/2
YAP/TAZ	Yes-associated protein/transcriptional coactivator with PDZ-binding motif
JAK/STAT	Janus kinase/signal transducer and activator of transcription
IFN-γ	Interferon-γ
IRF1	Interferon regulatory factor 1
COVID-19	Coronavirus disease 2019
SARS-CoV-2	Severe acute respiratory syndrome coronavirus 2
CRGs	Cuproptosis-related genes
NSCLC	Non-small cell lung cancer
LUAD	Lung adenocarcinoma
MA-ALI/ARDS	Malaria-associated acute lung injury/acute respiratory distress syndrome
Mtb	Mycobacterium tuberculosis
SRF	Serum response factor
NEDD4L	Neural precursor cell expressed developmentally downregulated 4-like
PVT1	Plasmacytoma variant translocation 1
SF1	Splicing factor 1
AARS1/2	Alanyl-tRNA synthetase 1/2
METTL16	Methyltransferase-like 16
CAP	Community-acquired pneumonia
DBT	Dihydrolipoamide branched chain transacylase E2
DLD	Dihydrolipoamide dehydrogenase
GCSH	Glycine cleavage system protein H
LIPT1	Lipoyltransferase 1
LUSC	Lung squamous cell carcinoma
SCLC	Small cell lung cancer
PDHA1	Pyruvate dehydrogenase E1 subunit alpha 1
GPRIN2	G protein-regulated inducer of neurite outgrowth 2
NLRP3	NOD-like receptor thermal protein domain associated protein 3
GLS	Glutaminase
MTF1	Metal regulatory transcription factor 1
PDHB	Pyruvate dehydrogenase E1 subunit beta
CDKN2A	Cyclin dependent kinase inhibitor 2A
